# Cheap Office Sign

**Published:** 1875-09

**Authors:** 


Cheap Office Sign—Imitation Ground Glass that Steam will not Destroy.—Put a piece of putty in muslin, twist the fabric tight, and tie it into the shape of a pad; well clean the glass first, and then putty it all over. The putty will exude sufficiently through the muslin to render the stain opaque. Let it dry hard, and then varnish. If a pattern is required, cut it out in paper as a stencil; place it so as not to slip, and proceed as above, removing the stencil when finished. If there should be any objection to the existence of the clear spaces, cover with slightly opaque varnish.—*Bost. Jour. Chem.*

It is not always safe for the laity to prescribe medicine. A lady writes to the *Courier-Journal* that her husband having heard that whisky was good for a snake bite, has been using it ever since a cow was bit, last spring, though the poor thing died, in spite of it, six weeks ago.—*Med. Surg. Reporter.*

